# The Apathy in Dementia Methylphenidate Trial 2 (ADMET 2): study protocol for a randomized controlled trial

**DOI:** 10.1186/s13063-017-2406-5

**Published:** 2018-01-18

**Authors:** Roberta W. Scherer, Lea Drye, Jacobo Mintzer, Krista Lanctôt, Paul Rosenberg, Nathan Herrmann, Prasad Padala, Olga Brawman-Mintzer, William Burke, Suzanne Craft, Alan J. Lerner, Allan Levey, Anton Porsteinsson, Christopher H. van Dyck, the ADMET 2 Research Group, Jacobo Mintzer, David Clark, Debra Battjes-Siler, Stan Smith, Courtney O’Neil, Nicole Stocking, Roberta W. Scherer, Lea Drye, Jamie Perin, David Shade, Jennifer Jones, Stephanie Holland, Alicia Wentz, Shumon Chattopadhyay, Bethany Grove, Stephanie Herrera, Kristen Kaiser, Andie Lears, April Broadnax, Aisha Mohammed, Laurie Ryan, Alvin McKelvy, Cerise Elliott, Kenneth Rockwood, Steve Edland, Raymond Scott Turner, Prasad Padala, Debbie Hodges, Nicole Jackson, Kalpana Padala, William Burke, Dawn Batchuluun, Anna Burke, Michele Grigaitis-Reyes, Marjoire DiLise-Russo, Shelia Vadovicky, Susan Favaro, Mary Lou Hernandez, Lynn Autry, Nicole Hoffmann, Rebecca Sanback, Lazaro Martinez Lujan, Elena Young, Alan Lerner, Susie Sami, Marianne Sanders, Parianne Fatica, Maria Gross, Ethan Gore, Supriya Mahajan, Allan Levey, James Lah, Tamara Attis, Janet Cellar, Chad Hales, Margaret Walker, Susan Peterson-Hazan, Erin Bolles, Erin Carter, Paul Rosenberg, Chris Marano, Jasmine Dixon, Sarah Lawrence, Meghan Schulz, Haroon Burhanullah, Anton Porsteinsson, Susan Salem-Spencer, Kim Martin, Audrey Rice, Nancy Kowalski, Michelle Cervello, Melanie Keltz, Kaitlyn Lane, Asa Widman, Olga Brawman-Mintzer, Jacobo Mintzer, Anthony Awkar, Abigail O’Connell, Arthur Williams, Sheila Howland, Alex Hails, Dennis Orwat, Nathan Herrmann, Krista Lanctôt, Abby Li, Damien Gallagher, Adam Dinoff, Danielle Vieira, Jennifer Bray, Eleenor Abraham, Suzanne Craft, Alecia Jenkins, Deborah Dahl, Samantha Rogers, Bonnie Sachs, Emily Mann, Edward Shaw, Kaycee Sink, Erin Caulder, Magie Jefferson, Camilla Martin, Kelsey Shore, Christopher H. van Dyck, Emily Kemp, Hannah Michalak, Melinda Becker, Erika Pugh, Tyler Godek, Martha MacAvoy, Susan Good, Megan Ebner, Julia McDonald, Srinath Ramanan, Adam Mecca, Oliver Lu

**Affiliations:** 1 Johns Hopkins University Bloomberg School of Public Health, Baltimore, MD USA; 2 Medical University of South Carolina and Ralph H. Johnson Veterans Administration Medical Center, Charleston, SC USA; 3 Sunnybrook Research Institute, Toronto, ON Canada; 4 Johns Hopkins University School of Medicine, Baltimore, MD USA; 5 University of Arkansas for Medical Science, Central Arkansas Veterans Healthcare System, Little Rock, AR USA; 6 Banner Alzheimer’s Institute, Phoenix, AZ USA; 7 Wake Forest University School of Medicine, Winston-Salem, NC USA; 8 University Hospitals – Case Western Reserve University, Cleveland, OH USA; 9 Emory University, Atlanta, GA USA; 10 University of Rochester, Rochester, NY USA; 11 Yale University, New Haven, CT USA

## Abstract

**Background:**

Alzheimer’s disease (AD) is characterized not only by cognitive and functional decline, but also often by the presence of neuropsychiatric symptoms. Apathy, which can be defined as a lack of motivation, is one of the most prevalent neuropsychiatric symptoms in AD and typically leads to a worse quality of life and greater burden for caregivers. Treatment options for apathy in AD are limited, but studies have examined the use of the amphetamine, methylphenidate. The Apathy in Dementia Methylphenidate Trial (ADMET) found that treatment of apathy in AD with methylphenidate was associated with significant improvement in apathy in two of three outcome measures, some evidence of improvement in global cognition, and minimal adverse events. However, the trial only enrolled 60 participants who were followed for only 6 weeks. A larger, longer-lasting trial is required to confirm these promising findings.

**Methods:**

The Apathy in Dementia Methylphenidate Trial 2 (ADMET 2) is a phase III, placebo-controlled, masked, 6-month, multi-center, randomized clinical trial targeted to enroll 200 participants with AD and apathy. Participants are randomly assigned 1:1 to 20 mg methylphenidate per day prepared as four over-encapsulated tablets or to matching placebo. The primary outcomes include (1) the mean difference in the Neuropsychiatric Inventory Apathy subscale scores measured as change from baseline to 6 months, and (2) the odds of having a given rating or better on the modified AD Cooperative Study Clinical Global Impression of Change ratings at month 6 compared with the baseline rating. Other outcomes include change in cognition, safety, and cost-effectiveness measured at monthly follow-up visits up to 6 months.

**Discussion:**

Given the prevalence of apathy in AD and its impact on both patients and caregivers, an intervention to alleviate apathy would be of great benefit to society. ADMET 2 follows on the promising results from the original ADMET to evaluate the efficacy of methylphenidate as a treatment for apathy in AD. With a larger sample size and longer follow up, ADMET 2 is poised to confirm or refute the original ADMET findings.

**Trial registration:**

ClinicalTrials.gov, NCT02346201. Registered on 26 January 2015.

## Background

Alzheimer’s disease (AD) is a growing public health problem with a global burden expected to exceed 80 million cases by 2040 [1]. This disease negatively impacts patients and families both emotionally and economically [2], with societal costs at about US$236 billion per year in the USA alone in 2016. Although cognitive and functional decline define AD, neuropsychiatric symptoms, such as agitation, delusions, hallucinations, depression, sleep disturbance, and problem behaviors, afflict almost all patients [3]. These symptoms lead to worse quality of life, greater disability, accelerated cognitive or functional decline, greater burden on caregivers, earlier institutionalization, and accelerated mortality [2]. Apathy is one of the most prevalent neuropsychiatric symptoms in AD [4, 5]. Clinically significant apathy is defined as a loss of will and initiative, lack of interest in activities, lack of productivity, and limited affective response to positive or negative events [6] and that is present for at least 4 weeks [7]. Apathy has been reported to affect more than half of people with dementia [8] and has devastating effects on the quality of life for both patients with AD and their caregivers. Patients suffering from apathy experience decreased motivation, relying heavily on caregivers to initiate and oversee daily activities. Those caregivers who lack an understanding of apathy as a syndrome may misinterpret apathetic patients as insensitive and uncaring [9] and report significant levels of distress and fewer positive experiences associated with caregiving than caregivers of non-apathetic patients with AD [10, 11]. Greater caregiver distress is linked with increased service utilization and accelerated institutionalization [12], which in turn creates a significant financial burden [13, 14]. Most notably, of all the neuropsychiatric symptoms apathy is the only symptom with high prevalence and marked persistence over the course of dementia [15]. Therefore, the management of apathy is a major priority in caring for patients with AD and reducing its public health burden.

There are no proven interventions to treat apathy in AD, but the use of catecholaminergic agents for the treatment of apathy is a promising and feasible approach to repurposing available medications for this purpose. This approach is based on the understanding that motivated behaviors rely not only on the dopaminergic mesolimbic brain reward system [16] but on newly evolved prefrontal cortical circuits that degenerate in AD, and where methylphenidate enhances both noradrenergic and dopaminergic signals to strengthen function [17]. Evidence for the use of catechoaminergic agents comes from case reports and small open-label studies in non-demented populations [18–21]. Short-acting methylphenidate has been one of the most studied catecholaminergic compounds in the elderly and presents a good safety profile [22]. It is well-tolerated during clinical use for the treatment of attention deficit hyperactivity disorder in children and young adults, the current indication approved by the Food and Drug Administration.

Data on the use of methylphenidate for the treatment of apathy in AD are sparse, but supported by case reports of methylphenidate for the treatment of apathy among adults and elders with major depression [23, 24], Parkinson’s disease [25], stroke [14], and in one instance for AD [26], and an open-label trial of methylphenidate in vascular dementia [19]. Additional evidence is provided by two pilot randomized clinical trials. The first randomized, placebo-controlled trial of methylphenidate for the treatment of apathy in AD was a small cross-over study that suggested that methylphenidate is modestly effective in most patients with AD [27–29]. The second, larger trial, the Apathy in Dementia Methylphenidate Trial (ADMET) found that methylphenidate treatment of apathy in AD was associated with significant improvement in two of three efficacy outcomes, a suggestion of an improvement in global cognition, and minimal adverse events [30, 31]. These data suggest that methylphenidate might be a safe and efficacious treatment for apathy in AD, although both randomized trials were small and had short follow up. To clarify the clinical efficacy of methylphenidate for apathy in AD more precisely, we are conducting a larger, longer trial with more robust measures: the Apathy in Dementia Methylphenidate Trial 2 (ADMET 2). ADMET 2 is a phase III, placebo-controlled, masked, 6-month, multicenter randomized clinical trial sponsored by the National Institute of Aging (NIA) involving 200 participants with AD. The trial is designed to examine the efficacy and safety of methylphenidate as treatment for clinically significant apathy in participants with AD, where efficacy will be measured by looking at changes in apathy and cognition. ADMET 2 will enroll participants from real-world settings and examine the effects of methylphenidate on apathy, cognition, cost-utility, and safety.

## Methods/design

The primary objective of ADMET 2 is to examine in a masked, placebo-controlled randomized trial the efficacy of methylphenidate for the treatment of clinically significant apathy in participants with AD. Additional objectives are to examine the cognitive effects, safety, and cost-effectiveness by assessing quality of life and economic assessment from baseline to 6 months. These assessments will be administered at baseline and at in-person follow-up visits, held monthly until 6 months after randomization. ADMET 2 is registered at ClinicalTrials.gov (NCT02346201) and is funded by the NIA, National Institutes of Health (NIH). The Sponsor has no role in study design, data collection or management, or writing of the final reports.

### Organization

Personnel in ADMET 2 will include a chair’s office (CO), coordinating center (CC), ten clinical centers and various committees. Primary decision-making bodies are the Executive and Steering committees, while data and safety monitoring will be conducted by an independent Data and Safety Monitoring Committee (DSMC). Additional committees are those on training and certification, recruitment, quality assurance, publication and presentation, ancillary studies, and policy and protocol. The CO is responsible for coordinating study meetings, training clinical staff on cognitive and apathy assessments, and preparing recruitment materials. The CC is responsible for maintaining data integrity, managing adverse event reporting, communicating protocol modifications, and preparing all materials associated with study meetings.

Current clinical sites, responsible for recruiting, treating, and following study participants include: Emory University, Atlanta, GA, USA; Johns Hopkins School of Medicine, Baltimore, MD, USA; Roper–St. Francis Healthcare, Charleston, SC, USA; Sunnybrook Research Institute, Toronto, ON, Canada; University Hospitals-Case Medical Center, Cleveland, OH, USA; University of Arkansas, Little Rock, AR, USA; University of Rochester, Rochester, NY, USA; Wake Forest University, Winston-Salem, NC, USA; and Yale University, New Haven, CT, USA. Banner Alzheimer’s Institute, Phoenix, AZ, USA discontinued active participation after 2 years. The use of diverse centers will promote representation from ethnic minority groups.

ADMET 2 aims to enroll 200 participants with clinically significant apathy from clinical centers. Participants will either be outpatients with AD, recruited from clinical settings at the study centers or residents of nursing homes or assisted living facilities. Inclusion criteria include those related to the presence of AD, apathy, mild to moderate cognition, and consent. The allowable range of cognitive impairment is as broad as possible to establish dementia diagnosis, but allow sufficient cognition for quantification of cognitive and apathy symptoms. The need for treatment for apathy is determined by the study physician when a patient has symptoms that are significant enough to require medication and “routine clinical care” has not resulted in improvement. The exclusion criteria were developed primarily to address patient safety, including known contraindications to the use of methylphenidate. Specifically, individuals with conditions that are contraindicated for methylphenidate use will be excluded as will persons with clinically significant agitation, hallucinations, or delusions. Specific eligibility criteria are itemized as shown below.

**Table:** 

**Eligibility criteria** Inclusion criteria
• Possible or probable Alzheimer’s disease (National Institute of Neurological and Communicative Disorders and Stroke - Alzheimer’s Disease and Related Disorders Association (NINCDS-ADRDA) criteria), with Mini-Mental State Exam (MMSE) score of 10–28 inclusive
• Clinically significant apathy for at least 4 weeks for which either
- the frequency of apathy as assessed by the Neuropsychiatric Inventory (NPI) is “Very frequently”, or
- the frequency of apathy as assessed by the NPI is “Frequently” or “Often” AND the severity of apathy as assessed by the NPI is “Moderate” or “Marked”’
• A medication for apathy is appropriate, in the opinion of the study physician
• Provision of informed consent for participation in the study by potential participant or surrogate (with participant assent if the potential participant is unable to provide informed consent) and caregiver
• Availability of caregiver, who spends greater than 10 hours a week with the potential participant and supervises his/her care, to accompany the participant to study visits and to participate in the study
• Sufficient fluency, of both the potential participant and caregiver, in written and spoken English to participate in study visits, physical exams, and outcome assessments
• If female, women must be postmenopausal for at least 2 years or have had a hysterectomy
Exclusion criteria
• Currently meets criteria for Major Depressive Episode, by *Diagnostic Statistical Manual of Mental Disorder - IV (TR)* criteria
• Clinically significant agitation/aggression for which either
- the frequency of agitation/aggression as assessed by the NPI is “Very frequently”, or
- the frequency of agitation/aggression as assessed by the NPI is “Frequently” AND the severity of the agitation as assessed by the NPI is “Moderate”, or “Marked”
• Clinically significant delusions for which either
- the frequency of delusions as assessed by the NPI is “Very frequently”, or
- the frequency of delusions as assessed by the NPI is “Frequently”’ AND the severity of the delusions as assessed by the NPI is “Moderate”, or “Marked”
• Clinically significant hallucinations for which either
- the frequency of hallucinations as assessed by the NPI is “Very frequently”’, or
- the frequency of hallucinations as assessed by the NPI is ‘Frequently’ AND the severity of the hallucinations as assessed by the NPI is “Moderate”, or “Marked”
• Change to AD medications within the 30 days preceding randomization, including starting, stopping, or dosage modifications
• Change in anti-depressant (except for trazodone used for sleeping difficulties as described below) use within the 30 days preceding randomization or a period of time equal to 5 half-lives of drug, whichever period of time is longer
• Use of trazodone >50 mg or lorazepam >0.5 mg or for indications other than sleeping difficulties within the 30 days preceding randomization or a period of time equal to 5 half-lives of drug, whichever period of time is longer. Other benzodiazepines are prohibited in the past 30 days or within 5 half-lives, whichever period of time is longer
• Failure of treatment with methylphenidate in the past for apathy after convincing evidence of an adequate trial as judged by study physician
• Currently taking any amphetamine product, an antipsychotic, bupropion, or any medication that would prohibit the safe concurrent use of methylphenidate, including but not limited to monoamine oxidase inhibitors and tricyclic antidepressants within the 30 days preceding randomization or a period of time equal to 5 half-lives of drug, whichever period of time is longer
• Need for acute psychiatric hospitalization or is suicidal in the opinion of the study physician
• Significant communicative impairments that would affect participation in the clinical trial
• Central nervous system abnormalities (e.g., cerebral aneurysm), seizures (convulsions, epilepsy), Tourette’s syndrome or presence of motor tics, or abnormal electroencephalography
• Lack of appetite that results in significant unintentional weight loss as determined by the study physician in the last 3 months
• Uncontrolled hyperthyroidism
• Any cardiovascular or cerebrovascular abnormality deemed to be clinically significant by the study physician, tachycardia (heart rate ≥100 beats per minute), or uncontrolled hypertension (defined as medication non-compliance or past 3 months with a diastolic reading ≥105 mm Hg), at the time of screening
• Closed angle glaucoma or pheochromocytoma
• Women with childbearing potential
• Current participation in a clinical trial or study that may add significant burden or affect study outcomes
• Any condition that, in the opinion of the study physician, makes it medically inappropriate or risky for the potential participant to enroll in the trial, including, but not limited to, contraindication to treatment with methylphenidate

A certified study physician must evaluate the participant before randomization. The study physicians will be responsible for fully assessing whether the participant has any of the conditions listed as contraindications for the use of methylphenidate (e.g., closed angle glaucoma, hyperthyroidism, or serious unstable cardiovascular or heart rhythm). The presence of any contraindicated condition or medication will constitute an exclusion criterion for ADMET 2. Laboratory tests for the purpose of qualifying a participant as having AD (e.g., brain imaging, blood and urine test, etc.) may be obtained prior to entry per current clinical standards and guidelines, but will not be required. The baseline visit may take place across more than one day, but must be completed within the 3 weeks preceding randomization.

To allow for generalizability to the usual clinical practice, ADMET 2 will allow concomitant use of a broad range of medications. The only exceptions are the use of medications that would prohibit the safe concurrent use of methylphenidate (any amphetamine product, bupropion, monoamine oxidase inhibitors, and tricycle antidepressants) or that function as dopamine receptor antagonists (antipsychotics). Changes in Alzheimer's disease medications during the study will be allowed if the clinician supervising the participant’s care determines the change to be clinically required and the clinician believes that the medication will not cause or exacerbate the participant’s apathy. For the treatment of sleeping difficulties, trazodone up to 50 mg before sleep or lorazepam up to 0.5 mg before sleep may be used nightly, or as needed. Use of other benzodiazepines or hypnotics will be prohibited.

### Interventions

Participants will be assigned to one of two groups: methylphenidate plus a psychosocial intervention for the caregiver, or placebo plus psychosocial intervention for the caregiver. The psychosocial intervention, while administered to both the participant and the study caregiver, is targeted primarily at the caregiver. The target dose of methylphenidate is 20 mg per day, provided as two over-encapsulated tablets of 5 mg administrated orally twice a day, once in the morning and once in the afternoon. Participants assigned to methylphenidate will start on 10 mg daily (i.e., one over-encapsulated tablet of 5 mg twice a day) for 3 days. The dose is increased to 20 mg per day (i.e., two over-encapsulated 5 mg tablets taken twice a day) on day 4 and continues for 6 months. Participants assigned to placebo will also begin with two capsules per day and on day 4 will also begin taking four capsules a day similar to the methylphenidate group. To aid participants and caregivers, a telephone contact is expected on day 3 or immediately before the dosage increase at day 4, and at any time a dose adjustment is required for clinical purposes. If a participant experiences unacceptable side effects on four capsules per day (20 mg methylphenidate or placebo per day), the study physician may decrease the dosage to two capsules a day (10 mg methylphenidate or placebo per day). Caregivers are asked to monitor and administer treatments for participants unable to do so for themselves.

For participants experiencing onset of significant agitation or delusions during the course of the trial, the study drug dose can be reduced to one capsule twice a day (10 mg per day in the methylphenidate group). If symptoms persist after reducing the dose the study drug can be temporarily discontinued, and can be restarted only when and if symptoms have improved and the participant discontinues any medication(s) used to treat the symptoms. However, study drug may only be restarted once the participant has discontinued that medication for at least 30 days or 5 half-lives, whichever is longer. If the participant requires initiation or change in an existing antidepressant (i.e., selective serotonin receptor inhibitor (SSRI)) for treatment of depression any time during the study, the study drug should be temporarily stopped until the participant is on a stable dose of the SSRI for at least 30 days or 5 half-lives, whichever is longer. Adherence to the assigned treatment will be monitored via participant and/or caregiver interview at each study visit and via pill counts. Participants and/or caregivers will be asked to return all study bottles with any unused capsules at each visit, including all empty bottles.

Participants will be monitored monthly for signs or symptoms of adverse effects. Methylphenidate is relatively short-acting with a mean half-life of 3.5 hours in adults; thus, if unexpected adverse events emerge, the investigator can simply reduce or discontinue the use of the study medication. If the unexpected adverse events disappear, the participant will re-start the study medication under close clinical supervision, including inpatient care if necessary. If the symptoms continue, it will be concluded that it is unlikely that the symptoms are related to the study medication. In that case, the participant will be treated as deemed clinically appropriate by the treating physician. The continuation or discontinuation of the drug will be decided by the center investigator in conjunction with the treating clinician based on the risk benefit consideration for each individual participant. For this reason, there will be no “rescue medication” used in this study.

Because there is no 5 mg formulation of methylphenidate that is approved and available both in the USA and Canada, the Canadian site will use a different generic methylphenidate formulation than that used at the US sites. The two drugs have similar bioavailability based on information from the drug manufacturers. We chose this solution to allow for similar titration of methylphenidate and to allow for results that are more generalizable than using a formulation that is not approved in one of the countries.

In addition to the drug, a trained study clinician will provide a standardized psychosocial intervention modeled after the counseling strategies employed for all caregivers and participants in the previous AD trials [31–33]. The psychosocial intervention consists of a counseling session, education materials, and 24-hour availability for crises. Counseling with the caregiver (and participant if available) takes place at each study visit, lasts approximately 20–30 minutes, and includes the following elements:

- Review and adjustment of the participant and caregiver supportive care plans
- Emotional support and opportunity to ventilate feelings
- Counseling on specific caregiving skills
- Assistance with problem solving in specific issues that the caregiver brings to the sessions
- Answers to questions on the educational materials

The educational component covers AD, its clinical course, symptomatic behaviors, behavioral management of apathy, and expectations for medication treatments. This information is included in *The 36-hour day* [34] and *Dementia care guidelines for families* [2], which will be given to each caregiver. The caregiver will also be provided with 24-hour phone access to the study nurse or physician for assistance with crises that may arise after hours. The psychosocial intervention will not be administered when the participant resides in a nursing home or receives care from a paid caregiver, because the content has been developed for supporting dementia home care by family members.

### Recruitment

Each site will develop their own recruitment plans tailored for potential study candidates from the local AD population. Recruitment activities may include chart review, telephone interviews and screens, discussion with physicians, and recruitment in the clinic waiting areas. Potential sources at each clinical center may include participants from established outpatient clinics at central or satellite locations, residents of assisted living facilities affiliated with the clinics, referrals by local physicians, or potential participants recruited from targeted advertisements in local media. The CO will create a prototype set of recruitment materials (i.e., brochure, poster, press release, and newspaper ad), designed to be appropriate for diverse ethnic and racial groups. Clinical centers may use these materials as part of their recruitment activities, supplementing with additional materials as needed. All recruitment materials will be approved by the local institutional review board (IRB) and be in accordance with Health Insurance Portability and Accountability Act (HIPAA) regulations to protect confidentiality.

The CC will regularly monitor recruitment by sending weekly updates to the clinic on the status of recruitment overall and of each clinic. Recruitment efforts will also be regularly reported to the Executive Committee and the Data and Safety Monitoring Committee.

### Randomization and masking

The CC generated random treatment assignment schedules using a documented program in SAS 9.2 (Copyright © 2002-2008 by SAS Institute Inc., Cary, NC, USA). The randomization schedule was designed to yield an assignment ratio of one to one for the two treatment groups, stratified by clinical center and using a permuted block design with random block sizes. Study participants and clinical center personnel, but not a restricted set of personnel at the CC, will be masked to treatment assignments,. The CC also generated a list of randomly ordered medication identifiers, which is linked to the assignment schedule. Documentation of the randomization generation processes will be retained at the CC and be accessible only to authorized personnel.

Treatments will be assigned using an online program accessible to the clinical centers through the web-based data system. After the entry of specified pre-randomization data, and confirmation of eligibility, each enrolled participant’s ID will be irrevocably linked to the next unassigned treatment for that clinical center. The clinical centers will be directed to issue a coded medication bottle containing the proper assigned treatment from among those available at the center. The data system will check to prevent randomization of the same participant ID. At each subsequent in-person follow-up visit, clinical center personnel will re-enter the participant ID to request a coded medication bottle for the next month’s supply of study drug. No study drug will be issued at the 6-month follow-up visit.

Clinical centers may request an emergency unmasking from the ADMET 2 data system and must contact the CC immediately following an emergency unmasking. Optional unmasking may occur for each participant after 6 months of follow up and when all data collection is completed. The treatment assignment will be provided in a sealed envelope to the participant and/or caregiver, but not revealed to the ADMET 2 staff at the clinical centers.

### Outcomes

The primary outcomes in ADMET 2 will measure the change in apathy from baseline to 6 months and include (1) mean difference in change from baseline to 6 months in the Neuropsychiatric Inventory Apathy subscale (NPI apathy) [35] scores; and (2) odds of having a given rating or better on the Modified AD Cooperative Study - Clinical Global Impression of Change (mADCS-CGIC) [33] ratings at month 6. Although the original ADMET trial included the Apathy Evaluation Scale (AES) [36], we chose not to include this as a primary outcome in ADMET 2. The AES requires a notable degree of clinician judgment while interviewing the caregiver, making it difficult to implement consistently across multiple centers and multiple psychometricians. A secondary apathy outcome measure is the Dementia Apathy Interview and Rating (DAIR) [37] scale. Other secondary outcomes will include the AD Cooperative Study-Activities of Daily Living Scale (ADCS-ADL) [38], Dependence Scale [39], and Information on caregiver distress (NPI caregiver distress score). Cost-effectiveness will be measured using assessment of health-related quality of life (EuroQol (ED-5D-5 L)) [40], and resource utilization (Resource Utilization in Dementia Lite (RUD-lite)) [41]. A battery of cognitive tests will be assessed at baseline and at the in-person follow-up visits at 2, 4, and 6 month, including the Mini-Mental State Exam (MMSE) [42], Hopkins Verbal Learning Test – Revised (HVLT-R) [43], the Digit Span: The Wechsler Adult Intelligence Scale – Revised Digit Span sub-test [44], Trail Making Tests (A and B) [45], Action Verbal Fluency Test from the Parkinson’s Disease–Cognitive Rating Scale [46], Category Fluency Task-Animal Naming [47], and the Short Boston Naming Test [48]. These tests are described in detail in Table 1.

**Table 1:** Description of data collection instruments used in ADMET 2

Instrument	Domains measured	Scoring	Administration
A. Apathy			
Modified AD Cooperative Study-Clinical Global Impression of Change (mADCS-CGIC)	Change in apathy	Seven-point scale where 1 is “very much improved” and 7 is “very much worse”; a rating of 4 being “no change”	Based on interview with caregiver by independent, skilled, and experienced clinician
Neuropsychiatric Inventory (NPI)	Frequency and severity of neuropsychiatric symptom (apathy, agitation, delusions, hallucinations, depression, euphoria, aberrant motor behavior, irritability, disinhibition, anxiety, sleeping, and eating disorders)	Scores determined by multiplying frequency (scored from 1 to 4) and severity (scored from 1 to 3) with caregiver distress (scored 1 to 5) and adding caregiver distress (scored 1 to 5); total score is sum of the score for each of the 12 (range is from 0 to 144). Higher scores indicate greater frequency and severity of symptoms	Interview with caregiver by clinician
Dementia Apathy Interview and Rating (DAIR)	Used to discriminate between apathy from lack of interest due to personality traits and evaluates change in motivation, engagement, and emotional response since disease onset	Each of 16 items scored from 0 to 3, with apathy score a sum of all items administered, divided by the number of items completed. Total scores range from 0 to 3, with higher scores representing more apathy	Interview with caregiver by clinician
B. Function			
Dependence Scale	Assesses the degree of dependence or assistance needed by a participant	Scored from 0 to 15, with higher scores indicating an increased level of dependence	Based on interview with caregiver by clinician
Cooperative Study-Activities of Daily Living Scale (ADCS-ADL)	Measures the functional performance of patients with AD	Scale discriminates between the stages of severity of AD, from very mild to severely impaired	Based on structured interview of caregiver by clinician
C. Cost-utility			
EuroQol EQ-5D- 5 L	Quality of life domains of mobility, self-care, usual activities, pain/discomfort, and anxiety/depression	Each domain graded from level 1 (no problems) to level 5 (extreme problems)	Interview of participant and/or caregiver by clinician
Resource Utilization in Dementia-Lite (RUD-Lite)	Assesses resource utilization and includes questions on accommodation, informal care, community care, and hospitalizations	Presence or absence of resource use	Interview of participant and/or caregiver by clinician
D. Cognition			
Mini-Mental State Exam (MMSE)	General cognition	Scored from 0 to 30, with higher scores indicating higher cognitive functioning.	Administered by trained interviewer to participant
Hopkins Verbal Learning Test – Revised (HVLT-R)	Cognition: verbal learning, recognition, and delayed recall	Scored from 0 to 12, with higher scores indicating better performance	Administered by trained interviewer to participant
Digit Span: the Wechsler Adult Intelligence Scale–Revised Digit Span sub-test	Cognition: auditory attention and working memory	Separate scores are obtained for spans read forwards and backward from 0 to 9 for the number of digits correctly identified and for the longest span that is recalled.	Administered by trained interviewer to participant
Trail Making Tests (A and B)	Cognition: attention, executive function, and visuo-motor tracking	Time taken to complete the test with shorter time indicating higher cognitive functioning	Administered by trained interviewer to participant
Action Verbal Fluency Test from the Parkinson’s Disease–Cognitive Rating Scale	Cognition: executive function, working memory, and information processing speed	Score is the total number of unique verbs, with higher counts indicating less cognitive impairment.	Administered by trained interviewer to participant
Category Fluency Task-Animal Naming	Cognition: executive function, working memory, set shifting, and executive control	Scores are the number of animals verbalized, with higher counts indicating less cognitive impairment	Administered by trained interviewer to participant
Short Boston Naming Test	Cognition: expressive language	The minimum score is 0 and the maximum score is 15. Higher scores indicate better control of expressive language	Administered by trained interviewer to participant

*AD* Alzheimer’s disease

Safety will be assessed by measuring vital signs, electrolytes, electrocardiograms (ECGs), and the NPI at each in-person visit. Adverse events reports will be completed as required at each follow-up visit. These measures, chosen based on the known possible side effects of methylphenidate, will be assessed at scheduled study visits and include blood pressure, pulse, respiratory rate, electrolyte levels (sodium, potassium, chloride, bicarbonate, glucose, urea nitrogen, and creatinine), ECG, and weight. Any clinically significant change in one of these measures will be considered an adverse event. In addition, ADMET 2 has compiled a list of known side effects of methylphenidate that are monitored at scheduled visits by interview of participants and their caregivers. All serious adverse events (SAEs), as defined by the Food and Drug Administration, will be recorded with study physicians rating the severity and relatedness of the event and any associated medical care.

**Table:** 

**Known side effects of methylphenidate collected as adverse reactions in ADMET 2** Behavioral	
• Aggressive behavior or hostility	
• Agitation	
• Anxiety, nervousness, or tension	
• Depressed mood	
• Distractibility	
• Drowsiness	
• Hyperactivity	
• Impaired learning	
• Impulsivity	
• Insomnia	
• Libido changes	
Cardiovascular	
• Angina	
• Blood pressure changes	
• Cardiac arrhythmia or serious heart rhythm abnormalities	
• Palpitations	
• Peripheral vasculopathy (including Raynaud’s)	
• Pulse changes	
• Tachycardia	
• Vasculitis	
Gastrointestinal	
• Abdominal pain	
• Abnormal liver function	
• Anorexia	
• Decreased appetite	
• Nausea	
• Weight loss	
Hematologic	
• Anemia	
• Thrombocytopenic purpura	
Musculoskeletal	
• Arthralgia (joint pain)	
• Dyskinesia (abnormal movement)	
• Muscle stiffness or aching, muscle tenderness	
• Rhabdomyolysis	
Neural	
• Blurry vision or eyesight changes	
• Dizziness	
• Dry mouth	
• Headache	
• Numbness of fingers, toes, nose, ears, lips	
• Tics (motor or verbal)	
Other	
• Fever	
• Hair loss	
• Priapism	
• Serotonin Syndrome	
• Skin rash, redness, or inflammation	
• Urine color change or decreased output	
• Urticaria	

### Study visits

Follow up will include both scheduled and unscheduled visits and contacts. Scheduled follow up will include in-person visits scheduled at monthly intervals after randomization, telephone contacts for data collection (days 15, 45, and 75 after randomization), and telephone contact for dose adjustments (day 3 and as needed). Target dates for follow-up visits will be calculated from the date of randomization. At all scheduled in-person visits, study staff will review study procedures to verify ongoing consent, the interim medical history and current medications. Assessments completed at each in-person visit will include AD and safety assessments. At months 2, 4, and 6, study staff will also collect functional assessments and perform the cognitive battery, and at months 3 and 6, study staff will perform cost-utility assessments. Data collected at the telephone contacts will include interim medical history, compliance, and adverse events. The specific data instruments administered at each scheduled contact are shown in Table 2.

**Table 2:** Data collection by visit

Months from BL	BL	T1	F1	T2	F2	T3	F3	F4	F5	F6
	0	0.5	1	1.5	2	2.5	3	4	5	6
Procedures										
Consent	X	X	X	X	X	X	X	X	X	X
History, or interim history	X	X	X	X	X	X	X	X	X	X
Demographics	X	-	-	-	-	-	-	-	-	-
Review of inclusion/exclusion	X	-	-	-	-	-	-	-	-	-
Psychosocial intervention	X	-	X	-	X	-	X	X	X	X
Dispensing of study drug	X	-	X	-	X	-	X	X	X	-
Review visit schedule	X	X	X	X	X	X	X	X	X	X
Review of compliance	-	X	X	X	X	X	X	X	X	X
Assessments										
NPI	X	-	X	-	X	-	X	X	X	X
mADCS-CGIC	X	-	X	-	X	-	X	X	X	X
DAIR	X	-	X	-	X	-	X	X	X	X
ADL	X	-	-	-	X	-	-	X	-	X
Dependence Scale	X	-	-	-	X	-	-	X	-	X
Cognitive battery	X	-	-	-	X	-	-	X	-	X
EQ-5D-5 L	X	-	-	-	-	-	X	-	-	X
RUD-lite	X	-	-	-	-	-	X	-	-	X
Diagnostic criteria for apathy	X	-	-	-	-	-	-	-	-	-
Safety measurements										
Vital signs	X	-	X	-	X	-	X	X	X	X
Electrolyte panel	X	-	X	-	X	-	X	X	X	X
Adverse events	X	X	X	X	X	X	X	X	X	X
Review ECG	X	-	X	-	X	-	X	X	X	X

*BL* baseline visit, *F* scheduled follow-up visits, *T* scheduled telephone contact, *NPI* Neuropsychiatric Inventory, *mADCS-CGIC* modified AD Cooperative Study Global Impression of Change, *DAIR* Dementia Apathy Interview and Rating, *ADL* Activities of Daily Living, *EQ-5D-5 L* Euro Quality of Life, *RUD-lite* Resource Utilization in Dementia-Lite, *ECG* electrocardiogram

### Sample size

Power calculations were conducted for the two primary outcomes: (1) mean difference in change from baseline to 6 months in the NPI apathy subscale scores; (2) odds of having a given rating or better on the mADCS-CGIC ratings at month 6. Both calculations assumed a type I error rate of 0.025 to preserve an overall type I error rate of 0.05 over both primary comparisons.

The power and sample size for the NPI apathy outcome were determined with standard two-sample methods for comparing means using SAS 9.2 (Copyright © 2002-2008 by SAS Institute Inc., Cary, NC, USA). The sample size calculations assumed the difference in NPI apathy change scores (1.8 points) and standard deviation (3.2) of the change scores observed in the original ADMET study [31]. The difference in NPI apathy is similar to the difference between mean change in “moderate improvement” and “minimal improvement” or “no change” observed in the ADMET trial. A sample size of 200 participants will ensure greater than 90% power to detect a difference of 1.8 points in change on the NPI apathy scale even if 15% of the participants are lost before the 6-month visit.

The power and sample size for the mADCS-CGIC outcome were determined using the method of Whitehead [49] for proportional odds logistic regression implemented by the “popower” in the Hmisc package in R (R version 2.1.3.1, Copyright © by The R Foundation for Statistical Computing).

We assumed the overall proportions of ratings in each category from ADMET: 3.5% with marked improvement; 8.8% with moderate improvement; 29.8% with minimal improvement; 54.4% with no change; 3.5% with minimal worsening; and 0% with moderate or marked worsening. Assuming an odds ratio for better ratings in methylphenidate of 2.75 (about 25% smaller than the odds ratio observed in ADMET) and 200 participants, the study will have 90% power to detect a difference between treatment groups with 10% losses to follow up and greater than 85% power with 15% losses.

### Data collection and management

ADMET 2 staff will collect study data on paper data collection forms and enter data online using the ADMET 2 website (http://admet.org/Public/ADMETPublic.html). Names, addresses, and other such personal data of participants and caregivers will not be entered and will not be part of the central database. Data collected from study evaluations and interviews will be identified only by study ID codes, which include the pre-specified participant ID and a four-letter code assigned at eligibility evaluation. Caregivers will also be identified by a unique ID code. The study will employ double data entry and validation checks against criteria specified in the data dictionary, including checks for consistency with other responses, and completeness. Data errors and inconsistencies will be flagged during the data entry process. Security measures will include password protection on a need to know basis for ADMET 2 staff. The database will be backed up daily and weekly on high-capacity cassette tapes. Monthly backups will be stored to CD media.

### Statistical methods

All primary analyses will be based on the “intention to treat” principle. Initial analyses will be descriptive in nature, using means, standard deviations, and proportions to describe baseline characteristics of the sample for all participants combined, and by intervention group. We will assess comparability of all baseline factors among the randomly assigned groups using appropriate statistical methods. Pearson correlation coefficients (or Spearman rank correlation) will be calculated to assess the strength of the associations between covariates and outcome measures; those with potential for confounding will be considered for inclusion in secondary analyses involving regression models. As required by NIH policy, we will conduct valid subgroup analyses of the primary and adverse event outcomes by gender and race/ethnicity to determine if there are possible differences (i.e., interactions) in treatment effects.

The primary outcome of difference in change from baseline to six months in the NPI apathy subscale scores will be analyzed initially by a crude comparison of the difference in mean change scores from baseline to month 6 using the *t* test. Longitudinal analyses of NPI apathy scores to compare treatment groups over time will use a saturated means model (including indicators for each visit and each visit-by-treatment interaction) adjusting for randomization stratification (clinic) by creating a linear mixed-effects model with random intercept for each participant to account for multiple measurements over time. The primary comparison is difference in mean change from baseline to month 6. A transformation of the outcome data will be used, if needed, to meet model assumptions. We will conduct planned subgroup analyses to see if treatment effects differ for (1) The US sites versus the Canadian site to account for the different generic methylphenidate used at these sites, (2) participants who meet the proposed diagnostic criteria for apathy at baseline versus those who do not meet criteria, and (3) apathy severity at baseline. Planned sensitivity analyses include comparison of the rate of change in NPI apathy scores over 6 months using target and actual visit times, using a subset of adherent participants and comparison of the proportion of participants with greater than 30% reduction in NPI apathy scores at 6 months.

The primary outcome of ratio of the odds of being at or better than a given category on the mADCS-CGIC at month 6 will be analyzed using proportional odds logistic regression to compare the mADCS-CGIC ratings of change (ranging from “marked worsening” to “marked improvement”) at month 6 between the treatment groups. The categorical outcome on the mADCS-CGIC of each participant at month 6 measures each participant's overall apathy at the endpoint relative to that at the baseline visit on a 7-point scale (1 = marked improvement, 2 = moderate improvement, 3 = minimal improvement, 4 = no change, 5 = minimal worsening, 6 = moderate worsening and 7 = marked worsening) and will be compared by assigned randomization group. If the data do not meet the proportional odds assumption, the Wilcoxon rank-sum test will be used to compare the mADCS-CGIC ratings. We will perform the same subgroup analyses described for the change in NPI apathy score.

Safety outcomes will be analyzed by assessing the adverse events data collected at each in-person visit, including those identified using systematic, close-ended questions for known or expected side effects of methylphenidate and open-ended questions for unexpected side effects, and abnormal results of electrolyte or ECG results. The proportion of participants experiencing adverse events and SAEs will be compared between treatment groups using logistic regression or Fisher’s exact test, controlling for baseline imbalances if necessary. To assess the superiority of methylphenidate compared with placebo for effects on cognitive and functional outcomes, we will look at longitudinal change in the DAIR, ADCS-ADL, Dependence Scale, and cognitive sphere scores from baseline to month 6 using a mixed-effects model as described above. For the pharmaco-economic analyses, costs (direct, indirect, and caregiver as reported in the RUD-Lite) will be summed for each treatment arm and compared with nonparametric tests, as cost data are typically skewed. We will conduct a cost-utility analysis by comparing total costs in each treatment arm with summation of utilities derived from the EQ-5D-5 L assessment to generate a cost per quality-adjusted life year (QALY). This will provide an important assessment of the cost-effectiveness of the intervention as we anticipate that treating with methylphenidate versus placebo will be economically attractive (i.e., potentially cost-saving if improved apathy is associated with lower overall costs and superior levels of functioning and quality of life).

For study participants with missing outcomes, multiple imputation will be considered, and analyses will follow the plans described above and the combination rules for multiple imputation. To investigate sensitivity to missing values, study participants with and without missing values will be compared by background covariates, and any observed differences will be adjusted for in the analyses.

### Quality control and performance monitoring

All ADMET 2 staff will be certified prior to performing trial activities to document a minimum level of competency to perform the functions of that role. In addition, each person requesting certification must sign a personal assurance of integrity in the data collection process, have completed a human subject/ethics training course within the last 5 years, and sign and complete the International Committee of Medical Journal Editors (ICMJE) Uniform Disclosure Form for Potential Conflicts of Interest. Persons completing the cognitive battery will be required to have administered at least seven of each type of assessment before being certified to complete that assessment. Study investigators will also be required to complete a clinical center certification form documenting sufficient space, facilities, and personnel and the requisite ethical approval to conduct the study.

Centers will be monitored centrally on a regular basis for rate of enrollment, protocol deviations, number and proportion of missed visits and losses to follow up, completeness of data, percentage of data items requiring edit queries, and percentage of discrepancies found in audited data items. In addition, site visits will be made to each of the clinical centers early in the course of recruitment and at other points in time as needed or desired for quality assurance purposes. Periodic audits of subsets of the database will also be conducted, both through visits to the centers and through a remote auditing procedure. At on-site visits, participant data will be chosen for verification from source documentation. For the remote auditing procedure, the CC will periodically review participant form sets.

### Data monitoring

The ADMET 2 DSMC committee will review accumulating data to monitor participant safety and evaluate the efficacy of methylphenidate for the treatment of apathy. They will provide a summary report of recommendations to the study chair and the CC following each meeting, and to the Steering Committee and the NIA in their capacity as an advisory committee. The DSMC will have three members, each of whom will have a background in geriatrics and psychopharmacological research related to AD, and an *ex officio* NIA representative. Formal analysis of the primary outcome data will be conducted and presented to the DSMC when 50% (at least 100 participants) of the expected participants have been enrolled in the study. The DSMC will not have any formal stopping rule, but will regularly review and evaluate accumulating safety data and may recommend termination of the trial if the risks become unacceptable.

Adverse events and SAEs will be defined as used in the DIADS-2 study [32]. Center investigators will be responsible for appropriate medical care of participants during the study, in connection with study procedures, and for monitoring the safety of participants. A medical monitor designated by the study chair will provide consultation to all centers on medical monitoring. The medical monitor and the CC will be notified using a special SAE report form within one working day by the investigative center via email and telephone if any of the following events occur: death, hospitalization or prolongation of hospitalization, life-threatening events or events that involve a persistent or significant disability or incapacity, or an unexpected event. Data collected on SAEs will include the treatment provided, outcome, and presumed relationship to study drug and will be updated as new information becomes available; a narrative description also will be provided. CC personnel review the data, and following any necessary clarification, forward the report to all investigators for submission to IRBs and to the DSMC as part of their safety review. Other adverse events will include monitoring for clinically significant changes from baseline for the following: vital signs, weight loss greater than or equal to 7%, MMSE score; NPI score, specifically for an increase in neuropsychiatric symptoms other than apathy (e.g., hallucinations, delusion, etc., and abnormal electrolytes or ECG).

### Ethics

ADMET 2 will be conducted according to the ethical principles of the Declaration of Helsinki. This study received ethical approval from the IRBs of Johns Hopkins Bloomberg School of Public Health (for the CC and CO), and from IRBs of each clinical site. Both participant and caregiver (if required by the local IRB) will be required to provide consent to participate in ADMET 2. Capacity of the potential study participant to give consent will be assessed in clinical interviews by investigators trained in obtaining consent from decisionally impaired persons. Caregivers will provide consent for potential participants who are unable to provide consent; the participant will be asked to provide assent. If potential participants are able to provide informed consent, they will be asked to do so and their caregiver will co-sign the consent form as a witness. The process of obtaining consent and assent will be documented in every case. ADMET 2 clinical staff will continue to obtain assent at each subsequent visit or during implementation of study procedures to assure continuing informed consent on the part of the proxy, to maintain assent by the participants, and to assess capacity. Only individuals who can provide informed consent for themselves can be caregivers. Each caregiver will be asked to provide informed consent for participation as informant and also to provide data on themselves as caregivers in the course of the study. Caregiver informed consent will be required unless otherwise stated by the center’s local IRB.

### Dissemination policy

Authorship for all ADMET 2 manuscripts describing the main findings (i.e., comparisons of treatment groups) will follow hybrid corporate or group authorship format, naming “The ADMET 2 Research Group” as author, with individual investigators and clinical center staff acknowledged. Other manuscripts may have conventional authorship. The primary results will be published before presentation of the results at any conference. Decisions on timing, content, and conclusions of publications rest with the ADMET 2 Steering Committee, although some of these decisions will be delegated to a Publication Committee. No comparison data will be available prior to completion of the study and no clinic may publish data obtained from their clinic independently. Writing committees for ADMET 2 papers will include at least one representative from the study chair’s office, one from the CC, and other study group members based on interest and expertise. All ADMET 2 manuscripts will be submitted to journals complying with the NIH Public Access Policy, and all publications will be archived in PubMed Central as required by this policy. Presentations at conferences describing or presenting ADMET 2 results follow the same guidelines and require clearance by the Steering Committee.

### Public access to protocol and data

The CC will facilitate data sharing in accordance with the NIH Data Sharing Policy. Study data will be provided to all ADMET 2 investigators after data collection is complete. ADMET 2 data will become available to outside investigators at the conclusion of the trial and following publication of the main study findings as a limited use dataset with documentation. Study participants are informed about data sharing with external investigators in the consent forms. All outside investigators will be asked to sign a data use agreement to protect study participant confidentiality.

## Discussion

This report described ADMET 2, a randomized trial comparing 20 mg of methylphenidate with placebo for the treatment of apathy, one of the most prevalent neuropsychiatric symptoms of AD. ADMET 2 is planned as a larger, longer study following ADMET, a randomized trial in which methylphenidate was shown to reduce apathy in the Apathy Evaluation Scale and the mADCS-CGIC, but not in the NPI. In ADMET, methylphenidate was administered only for 6 weeks and had a study population of 60 participants. In contrast, ADMET 2 plans to enroll 200 participants and follow them for 6 months. ADMET 2 is testing the efficacy of methylphenidate in reducing apathy as measured by three different instruments: mADCS-CGIC, NPI and DAIR. In addition, because ADMET study findings suggested the possibility of an improvement in cognition, ADMET 2 will administer a battery of cognitive tests aimed at distinguishing whether the apparent improvement was in fact an increase in cognition or simply an increase in attention during cognitive testing. Given the prevalence of neuropsychiatric symptoms and especially apathy in AD and its impact on both patients and caregivers, an intervention to alleviate apathy would be of great benefit to society.

### Trial status

**Fig. 1:**
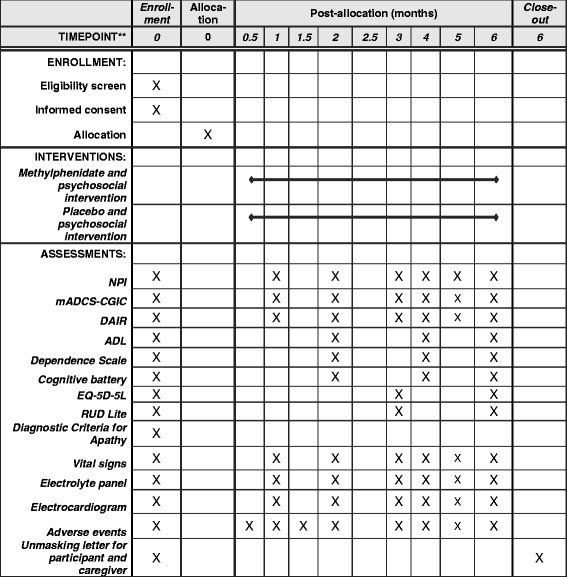
Standard protocol items: recommendation for interventional trials (SPIRIT) figure for the Apathy in Dementia Methylphenidate Trial 2. The cognitive battery includes the following assessments: Mini-Mental State Exam, Hopkins Verbal Learning Test – Revised, Wechsler Adult Intelligence Scale – Revised Digit Span sub-test, Trail Making Tests (A and B), Action Verbal Fluency Test from the Parkinson’s Disease–Cognitive Rating Scale, Category Fluency Task-Animal Naming, and the Short Boston Naming Test. *Abbreviations*: *NPI* Neuropsychiatric Inventory Apathy, *mADCS-CGIC* modified AD Cooperative Study Clinical Global Impression of Change, *DAIR* Dementia Apathy Interview and Rating, *ADL* Cooperative Study-Activities of Daily Living Scale, *EQ-5D-5 L* EuroQol 5D-5 L, *RUD-Lite* Resource Utilization in Dementia-Lite

This report describes the protocol, version 1.3, 02 May 2017 and describes the ADMET 2 protocol and adheres to the standard protocol items: recommendation for interventional trials (SPIRIT) reporting guidelines with attached checklist and figure (see Fig. 1).

ADMET 2 is currently recruiting study participants. The first study participant was randomized on January 2016. As of 1 October 2017, 77 of 200 study participants have been enrolled. The targeted end date for recruitment is June 2018.

## Abbreviations

AD: Alzheimer’s disease
ADCS-ADL: Cooperative Study-Activities of Daily Living Scale
ADCS-mCGIC: modified Cooperative Study Clinical Global Impression of Change
ADMET: Apathy in Dementia Methylphenidate Trial
ADMET 2: Apathy in Dementia Methylphenidate Trial 2
CC: Coordinating center
CO: Chair’s office
DAIR: Dementia Apathy Interview and Rating
DSMC: Data and Safety Monitoring Committee
ECG: Electrocardiogram
HIPAA: Health Insurance Portability and Accountability Act
HVLT-R: Hopkins Verbal Learning Test – Revised
ICJME: International Committee of Medical Journal Editors
IRB: institutional review board
MMSE: Mini-Mental State Exam
NIA: National Institute of Aging
NIH: National Institutes of Health
NPI: Neuropsychiatric Inventory Apathy
RUD-Lite: Resource Utilization in Dementia-Lite
SAE: Serious adverse event
